# Grandmothers’ perspectives on the changing context of health in India

**DOI:** 10.1186/s13104-017-2583-z

**Published:** 2017-07-07

**Authors:** Solveig A. Cunningham, Susannah D. Gloor, Shailaja S. Patil

**Affiliations:** 1 Hubert Department of Global Health, Emory University, 1518 Clifton Road NE, Office 7045, Atlanta, GA 30322 USA; 2 Department of Behavioral Sciences and Health Education, Emory University, Atlanta, USA; 3 Department of Community Medicine, BLDE University Shri B.M.Patil Medical College, Vijayapura, India

**Keywords:** Chronic disease, Globalization, Grandmothers, India, Gender roles, Data collection

## Abstract

**Background:**

The prevalence of obesity and other chronic diseases is increasing in India and around the world. As globalization and social changes are believed to be at the root of these epidemiological changes, these factors must be better understood. This study engaged older adults to gain an important perspective on globalization and health.

**Methods:**

A free-list instrument and a structured survey were developed and used to gather data on changes in diet, activity, and women’s roles from ten grandmothers in rural India.

**Results:**

Grandmothers indicated that household chores and food preparation are less labor-intensive and time-consuming due to mechanization and the availability of prepared foods than a generation earlier. Families are more often eating food out, bringing prepared food home, and using ready-made food mixes; adolescents are continuing to eat meals at home, but now snack with friends outside the home more frequently.

**Conclusions:**

Using both a free-list instrument and a structured survey, grandmothers were able to provide insights about the changing context of dietary patterns and family roles arising with globalization that may be contributing to the rise in chronic disease.

**Electronic supplementary material:**

The online version of this article (doi:10.1186/s13104-017-2583-z) contains supplementary material, which is available to authorized users.

## Background

A recent focus of research has been the increase in overweight and obesity in less developed settings, including India [1–4]. This trend may be due to globalizing influences such as urbanization; the mechanization of laborious tasks; new sedentary activities involving televisions and computers; and the availability of cheap, energy-dense foods [5–8]. In India, this “nutrition transition” is the result of increased availability of meat, milk products, and fats and less consumption of traditional beans, lentils, and other plant-based foods [3]. In addition, the expansion of the global marketplace has introduced new foods altogether into Indian communities. For example, processed foods high in sugars and fats, such as soda, packaged snacks like Oreos, and Cadbury chocolates, are becoming more widespread [9]. This pattern may be due in part to the ways in which transnational food companies alter food availability in developing countries in four ways: more processed foods, more fast food outlets, more large supermarkets, and more food advertising and promotion [10]. Furthermore, fried foods like samosas and fried chicken “Manchurian” are traditional foods of North India but are now more available in smaller communities across the country [9, 11].

Additionally, sedentary work and pastimes are increasingly common. In a study conducted in the same study area, we found that, for adolescents, active pastimes were the most popular form of recreation, but that the majority of activities children reported doing were sedentary [12]. Sedentary behaviors, such as watching television may be linked with obesity, including in India [7]. The mechanization of certain household tasks and modes of transportation also decreases levels of daily physical activity and is associated with an increase in obesity prevalence [8].

There is limited empirical evidence to provide context for how such changes are actually taking place. One way to obtain a potentially informative perspective on this process of change is to engage older adults, who would have witnessed these changes first-hand. Grandmothers especially are stalwarts of the family who spent and continue to spend most of their time within the home. As a result, they may have important insights into how social changes relate to family nutrition and leisure activities. In addition, previous studies have indicated that grandmothers are important forces in shaping the family food environment and promoting children’s health [13–18].

To explore the possibilities for garnering a view of sociocultural changes in the context of globalization from older adults, we used two approaches to engage ten grandmothers as part of a larger research program in a remote middle-sized city in India. The focus was on aspects of the home environment relevant to child health from the perceptions of grandmothers about the changes they have witnessed in their families in terms of diet, activity patterns and gender roles in a globalizing community. In this report, we describe preliminary information about the changing environment as it may relate to child health from grandmothers’ perspectives, and review the potential feasibility of engaging grandmothers in future research.

This research is based in Bijapur District in the state of Karnataka in Southern India, a socio-economically under-developed area that is undergoing economic, social, and cultural changes associated with globalization. Bijapur City, the main urban center, has increased in population by 43% since 2001, to an estimated 326,360 [19]. While under-nutrition continues to be a problem, with 42% of children under the age of 3 years being underweight in Karnataka [20], there is concern that obesity and other chronic disease are emerging.

## Methods

This research was part of a larger study focused on the home environment and risks of unhealthy weight among adolescents. A representative sample of 407 school-going adolescents aged 13–16 years was selected from 3 government and 3 private schools in Bijapur city. A stratified random sample of 201 government school students (102 boys, 99 girls) and 206 private school students (105 boys, 101 girls) was drawn from school rosters. Information from the adolescents and from their mothers was collected about the adolescent, the family, and the home environment in 2012. The cohort was re-visited in 2013 and this study is part of the follow up survey. The BLDE (Bijapur Liberal Development Education Association) University Ethics Committee approved the human subjects protocol for this study; research was carried out in compliance with the Helsinki Declaration.

To recruit participants for this study, we created a list of all sampled households with grandmothers living in them based on information collected in the original survey (122 households of 407). Of these, in 64 households the adolescent was attending private school and in 58 the adolescent was attending public school. We took note of the type of school attended because research from India has indicated socioeconomic differences between families according to the type of school they access. Five households with grandmothers were selected from each list and the ten grandmothers were recruited via telephone after they agreed to participate. Grandmothers were included if they had lived with the present family at least 5 years. All grandmothers who were selected agreed to participate.

As we found no previous studies that engaged grandmothers to study sociocultural changes, an exploratory study was developed using a short structured survey and a free-list instrument (see Additional file 1). The structured questionnaire consisted of 27 close-ended, pre-coded questions and two open-ended questions. It focused on sociocultural characteristics of the grandmother’s current household and of the household in which she was the mother of adolescent children decades earlier. Respondents were asked to describe and to compare characteristics of their current and past household, including details of eating, activity practices and daily tasks.

Free-listing is an anthropological method used to learn about an unfamiliar cultural domain and to solidify where concentrated research efforts are warranted [21]. The method involves asking respondents to list as many items as they can in response to a particular concept and probing to encourage participants to mention all aspects that come to their minds. Data collection was conducted orally in respondents’ homes in the local language, Kannada, in teams of two in July 2013. Data were gathered from 10 grandmothers, from five grandmothers using structured questionnaires (3 from government and 2 from Private) and from five (3 from private and 2 from government) using the free-listing method. Through both methods, we inquired about topics that are expected, based on the literature, to be pertinent to globalizing changes in diet and lifestyle. The domains covered in freelists were household tasks, roles outside the home, food preparation, eating behaviors, and physical activity. For each domain, the grandmother was asked to compare with the time when their son or daughter were of the same age as the adolescent grandchild included in the research project. The free-list instrument began with the same demographic questions as the survey and included five open-ended questions about gender roles, food preferences and habits, and levels of physical activity in the current and past households.

Grandmothers’ responses on the two instruments were recorded and data were entered in Excel. We listed and tabulated frequencies. Items from the free-lists were tabulated and counted in each domain. Responses to the survey questions were tabulated individually and then cross-tabulated against each other.

## Results

Grandmothers were between 52 and 80 years old; six of them were widowed. Grandmothers had been living in their current living arrangements on average 7 years. Seven were living with their son’s family and were thus the paternal grandmothers of the family’s children, while three were maternal grandmothers living with their daughter and son-in-law. In this part of India, the cultural norm is for the paternal grandmother to stay with family; when older women have several sons, they may stay with their eldest son, their youngest son, or the son who is managing their ancestral property. A woman may stay with her daughter’s family if she is widowed and does not have a son to look after her; it is not uncommon for this maternal grandmother to be the sister of her own son-in-law.

Most households (9/10) belonged to the Hindu religion and the average household had 7 members. The majority of grandmothers (7/10) were in the families with average income levels: 10,000–30,000 INR, or 150–450 USD/month. Among grandmothers, most (9/10) were homemakers and one had work outside the home as an office assistant. Six grandmothers were illiterate and 4 had completed primary education (7th grade). There were no major socio-demographic differences between respondents to the 2 instruments, though grandmothers selected for the freelist instrument were slightly more often grandmothers of private-school adolescents and were also slightly older on average.

### Household tasks

Grandmothers reported that exclusively women were responsible for domestic tasks in their current households and in the household where they had been mothers of adolescent children. In the current household, the mother was always responsible for cooking (i.e. the grandmother’s daughter-in-law or daughter). Grandmothers whose grandchildren were in private schools (and generally wealthier) reported that their daughter-in-law and hired female servants or domestic helpers were responsible currently for house cleaning, laundry, and dishwashing, whereas grandmothers of public school children reported that these tasks were done by her daughter-in-law or granddaughter. Meal preparation, such as cleaning vegetables and grains, was done most frequently by the grandmother herself. In their past households when they had been mothers of adolescents, all grandmothers reported that they, as mothers, had been responsible for all of these tasks themselves. These patterns could suggest that household responsibilities may be shifting somewhat from being solely the responsibility of the mother to being the duties of other women, including hired servants. When asked in the free-list to name duties they themselves had done previously that are not done by mothers now, grandmothers reported (3/5) clothes washing, cooking (1/5), and caring for animals (3/5). Grandmothers (4/5) reported that mothers now have more time for leisure activities compared to in the past since household devices have mechanized the preparation of meals, resulting in less time required for cooking.

### Food preparation

Most grandmothers reported that food preparation has become less laborious now than in the past due to mechanization, availability of gas stoves instead of having to fetch firewood for cooking, and availability of prepared and packaged foods. The majority (4/5) indicated that pre-packaged mixes such as curry powders and masala pastes are more often used to prepare food in their household now than in the past, and that they more often purchase *atta* (traditional whole grain) flour now rather than grinding grains for flour at home. Almost all (4/5) grandmothers reported that they cooked traditional dishes and food items that are not cooked by their daughter-in-law or daughter for their grandchildren. One grandmother said, “earlier there was no grinder, we used to grind [grains] ourselves.” All respondents listed “mixer/grinder” as an appliance that is found in households now that was not found when they were mothers of adolescents. Other appliances frequently listed include refrigerator, gas stove, and pressure cooker.

### Eating patterns

Grandmothers (4/5) reported that their grandchildren ate more often at roadside eateries than did their children at the same age, with only one grandmother reporting no difference. Roadside eateries are stands that offer fried fast foods, such as samosas, *pani puri* (fried crisp stuffed with a spicy mixture), fried noodles, egg omelets, and fried cauliflower (“Manchurian”), many of which are traditional in Northern India, but not in this area. The increase in availability and consumption of these new and previously unknown foods is consistent with research showing that adolescents’ consumptions patterns in this region reflect a combination of global, non-local, and traditional foods, access, and preferences [9]. Grandmothers also reported that their grandchildren ate snacks outside the home more often than their children had at the same age, with two grandmothers reporting not having noticed a difference. Snacking outside the home can include buying bakery products and foods sold at convenience stores, such as chips, cookies, and candy. Pocket money increases adolescents’ access to snacking outside the home, and indeed grandmothers observed that pocket money for children is now a phenomenon: In freelists, one grandmother commented, “Earlier, children were not given pocket money, now it is more common practice” and when asked “do you ever give pocket money to your grandchildren to eat outside food,” 3/5 said that they do. When asked in the free-list what foods their grandchildren ate now that their children did not eat when they were adolescents, grandmothers listed ready-to-eat foods like instant noodles (Maggi), packaged Cheetos-like snacks (Kurkure), and bakery products like white bread, cake, and pizza. They also listed *idli* and *dosa*, which are traditional Indian foods but are more frequently prepared in this area now than in the past due to the ease of preparation that comes with mechanized kitchen tools. One grandmother said, “Previously, we did not eat rice as it was not grown locally, but now rice is eaten more.” Another said, “we used to eat jowar (millet) and corn flour rotis; now more wheat and rice is eaten”; this observation may be linked to government-subsidized distribution of these food items.

In the structured survey, all but one grandmother reported that their grandchildren more often ate with friends outside the home than did their children. All grandmothers indicated that the practices of eating outside home and of bringing snacks from outside to the home have increased in comparison to their family in the previous generation. However, all respondents also indicated that the frequency of eating family meals at home has remained same.

### Physical activity

When asked to list activities that their adolescent grandchildren did now that their children had not done when they were the same age, every grandmother reported watching television, and the majority (7/10) reported using the computer or playing mobile games. These reports suggest an increase in adolescents’ participation in sedentary activities in the current generation.

### Roles outside the home

In the current household, all grandmothers reported that their son or son-in-law (the father of the adolescents) worked outside the home. This is similar to their experiences when they were mothers of adolescents, when all but one had a husband who worked outside the home. The majority (3/5) of grandmothers also reported that currently their daughter-in-law or daughter worked outside the home. By contrast, only one grandmother reported that she herself had worked outside the home when she was the mother of an adolescent. When asked in the free-list to name tasks that mothers do now that the grandmother did not do when she was raising her children, grandmothers echoed the questionnaire findings that mothers now do “outside work” and “go outside for a job.” In addition, they listed (4/5) “going to school” and doing social activities such as going to the movies, parlor (beautician) or meeting with friends wearing modern clothes (2/5). These patterns are consistent with increasing participation of women outside the home, both in the formal workforce and in the social sphere.

## Discussion

This study explored a unique perspective of how globalization is changing health by learning from elders who have been observing changes in their communities over the course of their lifetimes. The research was implemented through a structured questionnaire and a free-list exercise conducted with a small sample of grandmothers in a remote developing city in southern India. Grandmothers were asked about norms and habits in their current household and in the household in which they themselves had been mothers of adolescents.

Grandmothers indicated that women continue to be solely responsible for household chores, which most often involve cooking meals, washing clothes and dishes, and cleaning grains, but the distribution of chores may be shifting from being solely the responsibility of mothers to also involving other women, such as hired helpers, daughters, and grandmothers. Grandmothers observed that household chores, particularly grinding and pounding foodstuffs for meals, sewing, washing clothes, gathering fuel, and cooking are changing due to mechanization and the availability of prepared foods, becoming less labor-intensive and time-consuming than a generation before. While mothers continue to be the primary providers of household labor and now participate more often in labor outside the home, they may also enjoy more leisure and educational activities. Understanding time allocation and its implications for family health in poorer settings is an important area of research for future studies.

With respect to children’s environments, grandmothers noted that adolescents are eating meals at home as much as they did a generation earlier, but that families are more often eating food out, bringing prepared food home, and using ready-made food mixes than they did previously. Grandmothers also reported that adolescents now snack with friends outside the home more frequently than in the previous generation. Grandmothers’ observations about changes in food preparation and eating patterns may be relevant to studies examining whether rising obesity levels result from increases in eating “junk” foods, eating outside the home, and expanding in sedentary activities [6–8, 22, 23].

Grandmothers offered insights into some of the changes in eating and activity in their families and in norms occurring over the past generation. They were eager to narrate their experiences as the traditional caretakers of the home and seemed to feel valued due to participation in this research. All grandmothers cooperated fully with both instruments. The structured survey was fast to administer, while the freelisting method was more time-consuming. However, the respondents enjoyed sharing their observations more freely with the latter method. All grandmothers participating in free-listing commented about changing diet patterns, especially more rice consumption and mechanization of food production, about the new trend of giving pocket money to adolescents, and about the expansion of leisure time of mothers today. Free-listing is a method that has promise for engaging older adults in non-Western settings.

This was an exploratory study with a small number of respondents and utilizing two instruments not previously validated. The participants were women co-residing with their grandchildren who were ages 15–18 years in 2013. They were not selected to be representative of older adults in our study community. As is the case more broadly with studies collecting retrospective data from older adults, the information was reflective of participants’ perceptions and narratives, but may not be a reliable source of information about actual changes and events. For example, it may be easier for grandmothers to remember what their grandchildren eat now compared to what their children ate many years ago. Their reports may be shaded by beliefs that they worked harder and did things better than their daughters-in-law and daughters do now, or that, in the past, things were done more “properly” than now; if that is the case, they may overstate changes or the extent of unhealthy behaviors now compared to the past.

Building on this exploratory research, future studies should use adequately-sized population-representative samples of older adults to develop provide insights into the themes on which we were only able to touch broadly here. One useful approach would be to expand and validate the structured instruments developed here to systematically explore differences in perceptions and experiences across genders, age groups, social strata, and rural and urban residence. Using qualitative methods, including focus groups to examine patterns both across families and within families, for example comparing responses of older men and women or of grandmothers and mothers would add further context and may be useful in mapping perceptions to actual experiences.

Studies have shown that grandparents play a role in grandchildren’s feeding and activity patterns, especially in Southeast Asian families living with multiple generations [24, 25.] A future step for research will be to expand the inclusion of grandparents in eliciting information about their roles in deciding grandchildren’s diet and activity patterns; asking grandparents about their perceptions of their adolescent grandchildren’s weight status would also add knowledge about the family context of weight and weight-related behaviors and about the changing social contexts of health.

## Conclusions

This study demonstrates that older adults can provide valuable information for contextualizing the social and home environments relevant for health. Information from questionnaires and free-lists with older adults can also help guide the development of longitudinal studies that would more directly measure normative and social changes. Such research can enhance understanding of the social context of global behavioral and health changes.

## Additional file

**Additional file 1.** Survey and free-list instruments used.

## Abbreviations

BLDE: Bijapur Liberal Development Education Association
INR: Indian rupees
USD: United States dollars
