# Supplementary material for: Grandmothers’ perspectives on the changing context of health in India
Source: BMC Res Notes. 2017 Jul 7;10:263. doi: 10.1186/s13104-017-2583-z (PMC5501012; doi:10.1186/s13104-017-2583-z)
Supplement: Supplementary file 1 — Additional file 1. Survey and free-list instruments used. [file 13104_2017_2583_MOESM1_ESM.zip › Grandmothers_freelist.pdf]

BLDE UNIVERSITY'S SHRI B.M. PATIL MEDICAL COLLEGE, BIJAPUR

# GLOBALIZATION AND HEALTH SURVEY

## FREELIST INTERVIEW – HOUSEHOLD APPROACHES TO EATING AND ACTIVITY

| PARTICIPANT'S IDENTIFICATION |
|---|
| Adolescent Identification ___________________________<br><br>**CIRCLE:**<br>Paternal or Maternal grandmother: Paternal Maternal |

| |
|---|
| DATE (DD/MM/YY): [__\|__]/[__\|__]/[__\|__]TIME (hh:mm): [__\|__]:[__\|__]<br><br>INTERVIEWER'S NUM: [__\|__\| |
| RESULT :1=Completed 2=Incomplete 3=Refused 4= Not available |

| | SUPERVISOR | ENTERED BY |
|---|---|---|
| INITIALS | ____________________ | ____________________ |
| DATE | ____________________ | ____________________ |

**BLDE University's Shri B.M. Patil Medical College**

This survey is being administered on behalf of BLDE University's Shri B.M. Patil Medical College. The purpose is to learn what grandmothers think about approaches to eating and activity now versus when they were younger. The information from this survey will be used to understand if approaches to food and activity in the household have changed.

We would like you to answer some written questions. There is no time limit. For each question, please give as many answers as you can.

The survey will last approximately 20minutes. You do not have to participate, but if you do your answers will be kept secret. If you do not want to answer any of the questions, you may skip those or stop the survey at anytime.

**(INTERVIEWER: ask the participant if they agree to participate. CIRCLE "agree" if they choose to participate in the exercise).**

AGREE TO PARTICPATE DO NOT WISH TO PARTICIPATE

**INTERVIEWER INSTRUCTIONS:**

1. Write participant answers as they are said in the language the student uses. These will be translated later.

2. Most things that the participant names will be one or two words, but some responses may be a phrase. This is fine. Please write down whatever the participant says. If the response is more than a few words, write down enough to get the main message of the response. Do not interpret what the participant said in your own words.

3. Do not stop the participant in the middle of responding.

4. If the participant stops or if **less than 8 items** are named, please:
   a. Repeat the last item, for example *"Pepsi, and…?"*

      OR

   b. Ask *"Can you think of anything else?*

      OR

   c. Ask *"Are there any other things like the ones you have already named?"* For example, if the participant only said pizza, ice cream, and Pepsi, then the interviewer should start with the first item listed and ask:
      i. *"Is there anything else like pizza?"* They should continue asking this

for all the items originally mentioned: *"Is there anything else like ice cream?" "Is there anything else like Pepsi?"*

5. If you have to prompt a participant with any of the above options, please note where you did it on the response sheet by drawing a line under the items listed without help.
    a. For example, if the participant says
        i. Pizza
        ii. Ice cream
        iii. Pepsi
        ________________________
        iv. Mirinda

    b. Please do this only after the first time prompted for each question. We want to know how many things the participants list easily for each question.

6. Never name something that the participant did not already list on their own. We want the participant's ideas, not the interviewer's ideas.

7. Encourage participants who are having trouble naming things by nodding or saying: *"Ok, anything else?"* after an item is listed.
    a. However, do not say things like *"Exactly"* or *"very good"* after an answer. Please do not be more excited for some answers than for others, as this may make the participant think that a certain type of response is preferred.

8. If participants ask whether they are answering correctly or if their answers are good, please tell them *"There are no wrong answers. We want to know what you think."*

| | |
|---|---|
| A1. What is your religion?<br>(only one response possible) | Hindu ----------------------------------------1<br>Muslim--------------------------------------2<br>Christian------------------------------------3<br>Jain------------------------------------------ 4<br>Buddhist------------------------------------ 5<br>None-----------------------------------------0<br>Other---------------------------------------96<br>Specify: ___________________<br><br>Refused-----------------------------------98 |
| A2. What is your approximate age? | __ __ years old |
| A3. How many people live in your current household (including yourself)? | __ __ people in household<br><br>Refused-----------------------------------98 |
| A4. Approximately what is your family's income each month?<br><br>**(Prompt: Please include income from all members who contribute to the household)**<br>**READ OPTIONS** | Less than 5000 Rs/month -----------------1<br>5001-10000 Rs/month----------------------2<br>10000-20000Rs/month---------------------3<br>20000 – 30000 Rs/month------------------4<br>More than 30000 Rs/month---------------5<br><br>Refused----------------------------------98<br>Don't know-------------------------------99 |
| A4a. If any Agricultural Income | Rs __ __ __ __ __per annum |
| A5. Have you ever had a paid job? | Yes-------------------------------------------1<br>No--------0 If No → Skip to A6<br><br>Refused----------------------------------98<br>Don't know-------------------------------99 |
| A5a. If Yes then what is that Job? | Professional.............................. 1<br>Govt. salaried employee............ 2<br>Private salaried employee……………...3<br>Business…………………………………4<br>Daily wage labourer(non agricultural)….5<br>House maid……………………………….6<br>Others specify…………………………96<br><br>Refused----------------------------------98<br>Don't know-------------------------------99 |

| | |
|---|---|
| A6. What is your husband's paid job? | Professional.............................. 1<br>Govt. salaried employee............ 2<br>Private salaried employee……………...3<br>Business…………………………………4<br>Daily wage labourer(non agricultural)….5<br>House maid……………………………….6<br>Others specify………………………….96<br><br>Refused----------------------------------98<br>Don't know-------------------------------99 |
| A7. What is your highest education level? | Illiterate-------------------------------------1<br>Primary school----------------------------2<br>High school--------------------------------3<br>College and above-----------------------4<br><br>Refused------------------------------------98<br>Don't know---------------------------------99 |

**(INTERVIEWER: read these instructions to the participant)**
The following questions are about household approaches to eating and activity. For each question, I want you to write as many answers as you can. I want you to be as specific as possible.

I will write your answers as you say them. Please remember that this is not a discussion between us but a way for me to listen to and record the specific items you say.

If you think of a new answer for one question after we have moved on to another question, then you may go back and tell me more answers for a previous question. If you do not understand any of the questions, I will help explain them.

You should do this survey by yourself. Please do not ask anyone else for answers. There are no wrong answers, and all of your answers will be kept secret.

Do you have any questions before we start?

1. What are some kitchen and home appliances that are found in a household in Bijapur today that were not found in households when you were raising your children? Please name as many **specific** home appliances as possible.

| | | | |
|---|---|---|---|
| 1 | 2 | 3 | 4 |
| 5 | 6 | 7 | 8 |
| 9 | 10 | 11 | 12 |
| 13 | 14 | 15 | 16 |

2. What household duties or tasks do women of your daughter's or daughter-in-law's age do now that you and your friends did not do when you were that age? Please name as many **specific** tasks or activities as possible.

| | | | |
|---|---|---|---|
| **1** | **2** | **3** | **4** |
| **5** | **6** | **7** | **8** |
| **9** | **10** | **11** | **12** |
| **13** | **14** | **15** | **16** |

3. What household duties or tasks do men of your son's age do now that your husband did not do when he was that age? Please name as many **specific** tasks or activities as possible.

| | | | |
|---|---|---|---|
| **1** | **2** | **3** | **4** |
| **5** | **6** | **7** | **8** |
| **9** | **10** | **11** | **12** |
| **13** | **14** | **15** | **16** |

4. What are some foods and beverages that your adolescent grandchildren eat now that your children **did not** eat when they were the age your grandchildren are currently? Please name as many **specific** foods and beverages as possible.

| | | | |
|---|---|---|---|
| **1** | **2** | **3** | **4** |
| **5** | **6** | **7** | **8** |
| **9** | **10** | **11** | **12** |
| **13** | **14** | **15** | **16** |

5. What are some foods and beverages that your children ate as an adolescent that your adolescent grandchildren **do not** eat now? Please name as many **specific** foods and beverages as possible.

| | | | |
|---|---|---|---|
| **1** | **2** | **3** | **4** |
| **5** | **6** | **7** | **8** |
| **9** | **10** | **11** | **12** |
| **13** | **14** | **15** | **16** |

6. What are some leisure time activities that your adolescent grandchildren do now that your children **did not** do when they were the same age? Please name as many **specific** activities as possible.

| | | | |
|---|---|---|---|
| **1** | **2** | **3** | **4** |
| **5** | **6** | **7** | **8** |
| **9** | **10** | **11** | **12** |
| **13** | **14** | **15** | **16** |

**ATTENTION: Please think again about the questions I asked and all of the answers you have given me. Can you think of any more things like the ones you already named? If you can think of any more, please tell me.**

| Thank you for your help! Is there anything else about this topic that you think we should know? Do you have any comments? |
|---|
| |

Time interview Ended:

| | | | |
|---|---|---|---|

| Interviewers Comments: |
|---|
| |
