# Supplementary material for: Grandmothers’ perspectives on the changing context of health in India
Source: BMC Res Notes. 2017 Jul 7;10:263. doi: 10.1186/s13104-017-2583-z (PMC5501012; doi:10.1186/s13104-017-2583-z)
Supplement: Supplementary file 1 — Additional file 1. Survey and free-list instruments used. [file 13104_2017_2583_MOESM1_ESM.zip › Grandmothers_module.pdf]

BLDE UNIVERSITY'S SHRI B.M. PATIL MEDICAL COLLEGE, BIJAPUR

GLOBALIZATION AND HEALTH SURVEY

GRANDMOTHER INTERVIEW

PARTICIPANT'S IDENTIFICATION

Adolescent Identification ____________________________

Name of the Grandmother _______

Paternal or Maternal grandmother: 1. Paternal 2.Maternal

(circle the option applicable)

Present marital status : 1. Married 2.Widow 3.Seperated/Divorced

Landline telephone number:____________________

Mobile number (Or household)______________________

DATE (DD/MM/YY): [__|__]/[__|__]/[__|__] TIME (hh:mm): [__|__]:[__|__]

INTERVIEWER'S NUM: [__|__|

RESULT :1=Completed 2=Incomplete 3=Refused 4= Not available

	SUPERVISOR	ENTERED BY
INITIALS	___________________	___________________
DATE	___________________	___________________

BLDE University's Shri B.M. Patil Medical College

My name is ___________________________.

This survey is being administered on behalf of BLDE University's Shri B.M. Patil Medical College. The purpose is to learn about adolescents' nutrition and physical activity in Bijapur. We would like to ask you some questions about the eating habits and physical activity of your grandchildren in your household. Your knowledge matters very much for our research. Participation is voluntary and all information you provide will be confidential and will be used for research purpose only.

The interview will last approximately 20 minutes. You do not have to answer any questions that you are uncomfortable with and you may stop the interview at any time

INTERVIEWER INSTRUCTIONS:

Only read response options for the questions for which you are instructed to do so below. NEVER read the categories DK (does not know), Refused, and NA (not applicable) that are listed on the questionnaire.

UNLESS OTHERWISE STATED, ONLY ONE ANSWER IS POSSIBLE

Part A: Family Demographics

First I would like to ask you about your family.

A1. What is your religion?	Hindu…………………………1<br>Muslim………………………..2<br>Jain……………………………3<br>Buddha………………………4<br>None of the above……………0<br>Others…………………………96<br>Specify………………………..<br>Refused…………………………98
A2. What is your age (approximate)?	___ _ yrs<br>Refused…………………………98<br>Don't know……………………..99
A3. Total number of family members residing with you , including you?	___ _ people<br>Refused…………………………98<br>Don't know……………………..99
A4. What is your total family income? (monthly)	< Rs. 5000/-…………………………1<br>Rs. 5000 to 10,000…………………...2<br>Rs. 10,000 to 20,000…………………3<br>Rs. 20,000 to 30,000………………...4<br>>Rs. 30,000…………………………5<br>Refused…………………………98<br>Don't know……………………..99
A4a. Agricultural income	Rs………………….. yearly

A5. Did you ever have job/occupation with income ?	Yes……………………….1<br>No………………………..0<br>**If no skip to A6.**<br>Refused…………………………98<br>Don't know……………………..99
A5a. If yes, which type of job/occupation?	Professional…………………..1<br>Govt. job……………………..2<br>Private………………………..3<br>Business………………………4<br>Daily wages…………………..5<br>House work…………………..6<br>Others , specify………………..96<br>Refused…………………………98<br>Don't know……………………..99
A6. What was your husband's occupation	Professional…………………..1<br>Govt. job……………………..2<br>Private………………………..3<br>Business………………………4<br>Daily wages…………………..5<br>House work…………………..6<br>Others , specify………………..96<br>Refused…………………………98<br>Don't know……………………..99
A7. What is your level of education ?	Illiterate…………………………1<br>Primary……………………………2<br>High school………………………3<br>College…………………………4<br>Refused…………………………98<br>Don't know……………………..99
A8. How many children you have (including Male and Female)	……………….. girls<br>………………...boys<br>Refused…………………………98<br>Don't know……………………..99
A9. How many grand children you have? (including Male and Female)	……………….. girls<br>………………...boys<br>Refused…………………………98<br>Don't know……………………..99
A10. Presently how many of your grandchildren stay with you?	……………….. Number<br>Refused…………………………98<br>Don't know……………………..99
A11. Do you take complete responsibility of looking after your grandchildren ?	Yes……………………1<br>No……………………2<br>Refused…………………………98<br>Don't know……………………..99

Part B: Now I will be asking about your present house conditions

B1. Who all stay with you presently?<br>(multiple options possible)	Daughter……………………….1<br>Son………………..2<br>Daughter in law…………….3<br>Son in law……………..4<br>Husband……………….5<br>Grand daughter………………6<br>Grand son…………………….7<br>House maid…………………..8<br>Other, specify……………………………9<br>Alone………………………….10<br>Refused-------------------------------------98<br>Don't know---------------------------------99
B2. Who all go outside for work?<br>(multiple options possible)	Daughter……………………….1<br>Son………………..2<br>Daughter in law…………….3<br>Son in law……………..4<br>Husband……………….5<br>Grand daughter………………6<br>Grand son…………………….7<br>Other, specify……………………………9<br>Refused-------------------------------------98<br>Don't know---------------------------------99
B3. Following are the list of people with their codes , write the code in front of the work they do in the house.<br><br>(multiple numbers can be written)<br><br>1- Daughter<br>2- Son<br>3- Daughter in law<br>4- Son in law<br>5- Husband<br>6- Grand daughter<br>7- Grand son<br>8- Maid<br>9- Other female<br>10- Other male<br>11- Me<br>Refused------------------------------------98<br>Don't know----------------------------------99	B3a. cooking----<br>B3b. house cleaning-------<br>B3c. washing clothes--------<br>B3d. washing utensils---------<br>B3e. cutting veg---------<br>B3f. monthly food purchase--------<br>B3g. gardening-----------<br>B3h. agricultural work-----------

C1. When your children were 10 – 15 yr old, where did you live ?	Outside Vijayapurdistrict…………..1<br>Vijayapur rural…2<br>Vijayapur urban….3<br>Others.. specify…………….4<br>Refused--------------------------------------98<br>Don't know----------------------------------99
C2. When your children were 10-15yr old, who all lived with you?<br>(multiple options possible)	Daughter…………….1<br>Son…2<br>Mother in law………..3<br>Father in law…………..4<br>Grand parents…………..5<br>Sister in law…………….6<br>Brother in law……………..7<br>Others, specify…………….8<br>Refused--------------------------------------98<br>Don't know----------------------------------99
C3. Out of the members who resided with you then, who went out for work/job ?<br>(multiple options possible)	Daughter…………….1<br>Son…2<br>Mother in law………..3<br>Father in law…………..4<br>Grand parents…………..5<br>Sister in law…………….6<br>Brother in law……………..7<br>Others, specify…………….8<br>Refused--------------------------------------98<br>Don't know----------------------------------99
C4. When you children were 10-15yr old who were doing what work? Identify from list below and fill infront of the type of work.<br>1- Daughter<br>2- Son<br>3- Daughter in law<br>4- Son in law<br>5- Husband<br>6- Grand mother<br>7- Grand father<br>8- Sister in law<br>9- Brother in law<br>10- maid<br>11- other female<br>12- other male<br>13- me<br>Refused------------------------------------98<br>Don't know----------------------------------99	B3a. cooking ____<br>B3b. House cleaning______<br>B3c. Washing clothes______<br>B3d. Washing utensils_______<br>B3e. Cutting veg_______<br>B3f. Grocery Shopping______<br>B3g. Gardening______<br>B3h. Agricultural work______

Part D: Now , I will be asking about diet and physical activity details of your family during the period , when your children were adolescents (10-15 years) and now , please recall and tell us

D1. If you compare the following food habits, can you tell in what way your grandchildren's eating habits have changed in comparison with the time when your children were adolescents ?(for each option only one answer is to be marked)

		Increased	Decreased	Same	Not observed
D1a	Eating street foods / fromroadside bakeries	1	2	3	4
D1b	Home made sweets (Traditional)	1	2	3	4
D1c	Eating outside home food	1	2	3	4
D1d	Eating outside with friends	1	2	3	4
D1e	All family members going to eat outside or bringing outside food	1	2	3	4
D1f	Inviting relatives or friends to house for lunch/dinner	1	2	3	4
D1g	Food prepared by Maid /domestic help	1	2	3	4

D2. Do your family members eat outside regularly?	Yes………..1<br>No……0<br>If no, …………………………**skip to D4**<br>Refused-------------------------------------98<br>Don't know----------------------------------99
D3. Do you go, with your family members to eat outside?	Every time……….1<br>Sometime………2<br>Rarely…………3<br>Donot go……………….4<br>Refused-------------------------------------98<br>Don't know----------------------------------99
D4. Do you prepare any special /traditional food for your grand children , (which is usually not cooked by their mother) ?	Yes………………..1<br>No…………………0<br>Refused-------------------------------------98<br>Don't know----------------------------------99

D5. If yes , name 3 food items which you prepare generally?	1…………………<br>2…………………<br>3………………..<br>Refused------------------------------------98<br>Don't know----------------------------------99
D6. Do you give pocket money to your grand children to eat outside?	Yes.1<br>No……0<br>Refused------------------------------------98<br>Don't know----------------------------------99
D7. Which type of food items do they buy from outside?	1…………………<br>2…………………<br>3………………..<br>Refused------------------------------------98<br>Don't know----------------------------------99
Part-E, Now I will be asking about the equipments and machines which your family uses on daily basis	
E1a. Which of the following equipments present in your house?<br>(please read the options slowly , multiple options possible)	Microwave………………………..1<br>Grinder……………………2<br>Mixer……………………….3<br>Vacuum cleaner……………..4<br>Washing machine………………..5<br>Car……………………6<br>Motor bike……………….7<br>Television……………8<br>Video player/ DVD player…………….9<br>Refused------------------------------------98<br>Don't know----------------------------------99
E2. If you compare the time when your children were adolescents (10-15 yr) and now , Do you find differences in following :	

	**Mark only one option for each**	**Increased**	**Decreased**	**Same**
**E2a**	Cooking related: Bringing ready to eat foods and spices , flour etc..	1	2	3
**E2b**	Hiring house maid/ domestic help	1	2	3
**E2d**	Food prepared by maid	1	2	3

Thank you for your participation and do you have anything else to tell us you can

Interviewer's notes:

Interview Finishing time :
